# Reintroduction of the endangered and endemic plant species *Cochlearia bavarica*—Implications from conservation genetics

**DOI:** 10.1002/ece3.3596

**Published:** 2017-11-15

**Authors:** Franziska Kaulfuß, Christoph Reisch

**Affiliations:** ^1^ Chair of Ecology and Conservation Biology University of Regensburg Regensburg Germany

**Keywords:** conservation, genetic variation, inbreeding, outbreeding, reinforcement, reintroduction

## Abstract

Population reintroduction is a common practice in conservation, but often fails, also due to the effects of inbreeding or outbreeding depression. *Cochlearia bavarica* is a strongly endangered plant species endemic to Bavaria in Germany, constantly declining since the late 1980s. Therefore, population reintroduction is intended. In this study, we analyzed genetic diversity within and genetic differentiation between all 32 remnant populations of the species in Swabia and Upper Bavaria using amplified fragment length polymorphisms. Our aim was to increase reintroduction success by providing data to avoid negative effects of inbreeding and outbreeding and to preserve the natural genetic pattern of the species. Genetic diversity within populations was low but similar to other rare and endemic species and varied strongly between populations but did not depend on population size. Our analysis revealed a strong geographic pattern of genetic variation. Genetic differentiation was strongest between Swabia and Upper Bavaria and at the population level, whereas differentiation between subpopulations was comparatively low. Isolation by distance and genetic differentiation was stronger among populations from Upper Bavaria than from Swabia. From the results of our study, we derived recommendations for a successful reintroduction of the species. We suggest using rather genetically variable than large populations as reintroduction sources. Moreover, the exchange of plant material between Swabia and Upper Bavaria should be completely avoided. Within these regions, plant material from genetically similar populations should preferably be used for reintroduction, whereas the exchange among subpopulations seems to be possible without a negative impact on genetic variation due to natural gene flow.

## 1 INTRODUCTION

The loss of plant species is a worldwide problem, mainly due to land use changes (Maurer, Weyand, Fischer, & Stöcklin, 2006; Poschlod, Bakker, & Kahmen, 2005) such as agricultural intensification (Storkey, Meyer, Still, & Leuschner, 2012) and abandonment of traditional management methods (Poschlod & WallisDeVries, 2002). The associated process of habitat fragmentation intensifies the loss of plant species (Fahrig, 2003; Schleunig, Niggemann, Becker, & Matthies, 2009), because small and isolated remnant populations suffer from a higher extinction probability (Matthies, Brauer, Maibom, & Tscharntke, 2004). The actual extinction rate is, therefore, 100–1,000 times higher than it would be naturally expected (Thuiller, 2007).

Population reintroduction, comprising reintroduction in the narrow sense, reinforcement, and translocation (Akeroyd & Wyse, 1995), is meanwhile a common practice in conservation to alleviate the proceeding loss of plant species. Generally, the aim of population reintroduction is to establish genetically variable populations, to increase gene flow (Akeroyd & Wyse, 1995; Betz, Scheuerer, & Reisch, 2013; Godefroid et al., 2011) and to minimize the probability of population extinction (Vergeer, van den Berg, Roelofs, & Ouborg, 2005).

However, population reintroduction is a challenge and often fails (Godefroid et al., 2011). One main reason for the lack of success is the origin of the plant material used for reintroduction, especially when reintroduced plants or seeds derive from small populations or only from a few individuals (Godefroid et al., 2011). Small populations are less attractive for pollinators (Agren, 1996; Aizen & Feinsinger, 1994; Kunin, 1997), which reduces cross‐pollination and increases self‐fertilization or mating with related individuals (Van Treuren, Bijlsma, Ouborg, & Kwak, 1994). Using plant material from small populations with limited genetic variation may increase indeed the census population size but even reduce effective population size (Friar, Ladoux, Roalson, & Robichaux, 2000; Robichaux, Friar, & Mount, 1997). Reintroduced populations may, therefore, suffer from inbreeding depression (Frankham, Ballou, & Briscoe, 2002; Friar et al., 2000; Robichaux et al., 1997).

Similar results can be evoked when reintroduced populations are founded with only a few individuals. Genetic variation of the reintroduced population may be reduced due to this founder effect (Vergeer et al., 2005). Furthermore, genetic drift may cause the random loss of alleles, increasing homozygosity and the fixation of deleterious alleles (Ellstrand & Elam, 1993; Young, Boyle, & Brown, 1996). Both, inbreeding and genetic drift, result in decreased genetic diversity and fitness (Booy, Hendriks, Smulders, Van Groenendael, & Vosman, 2000; Charlesworth & Charlesworth, 1987; Ouborg, Vergeer, & Mix, 2006; Young, Petersen, & Clary, 2005), and populations may thus lose their ability to adapt to changing environmental conditions (Booy et al., 2000; Heywood, 1991; Reed, Lowe, Briscoe, & Frankham, 2003).

Moreover, the success of population reintroduction may be limited due to the adaptation of populations to the environmental conditions of their habitat. It has been demonstrated previously that ecological differences among habitats result in different local adaptations or ecotype development (Becker, Colling, Dostal, Jakobsson, & Matthies, 2006; Joshi et al., 2001; Leimu & Fischer, 2008; McKay, Christian, Harrison, & Rice, 2005; Reisch & Poschlod, 2009). Mixing different genotypes adapted to specific habitat conditions can result in the erosion of coadapted gene complexes (Frankham et al., 2002). Local adaptations get lost, and outbreeding depression may result in decreased fitness and performance of the populations (Bischoff et al., 2006; Fischer & Matthies, 1998; Keller, Kollmann, & Edwards, 2000; Krauss, Zawko, Bussell, Taylor, & Hood, 2005; Mijnsbrugge, Bischoff, & Smith, 2010; Montalvo & Ellstrand, 2000, 2001), which may consequently decrease reintroduction success.

*Cochlearia bavarica* Vogt is a rare, endemic, and endangered plant species comprising a limited number of small and isolated populations (Fischer, Hock, & Paschke, 2003). The species occurs in only two regions of Bavaria, and the number and size of populations constantly declined since the late 1980s due to changes in land use, habitat loss, and fragmentation (Fischer et al., 2003). *Cochlearia bavarica* has, therefore, been included in the “German National Strategy on Biodiversity” and in two large conservation projects (“Wildpflanzenschutz Deutschland” and “Löffelkraut & Co”). Within these projects, it is intended to maintain and develop populations by protecting and restoring natural habitats of *C. bavarica*. Furthermore, it is purposed to augment small populations by population reinforcement and to reduce the loss of populations by population reintroduction.

The genus *Cochlearia* and its species already have been in the focus of many plant systematic and conservation studies (Brandrud, 2014; Cires, Samain, Goetghebeur, & Prieto, 2011; Koch, Dobeš, Bernhardt, & Kochjarová, 2003; Olsen, 2015; Paschke, Abs, & Schmid, 2002a). In this study, we analyzed the genetic diversity and differentiation among populations of *C. bavarica*. Our aim was to increase the success of future population reintroduction and reinforcement, by providing data to avoid negative effects of inbreeding and outbreeding and to preserve the natural genetic pattern of the species. In this context, the following questions were addressed: (1) How large is genetic diversity within populations and genetic differentiation among populations of *C. bavarica*? (2) Which populations may serve as potential sources for population reinforcement of small populations facing extinction or population reintroduction? (3) Is it possible to draw general conclusions for the reintroduction of *C. bavarica*?

## 2 METHODS

### 2.1 Species description

*Cochlearia bavarica* Vogt is endemic to Bavaria with a narrow distribution in Swabia and Upper Bavaria (Abs, 1999). The species is more frequent in Swabia than in Upper Bavaria and originated from hybridization of *Cochlearia pyrenaica* DC. and *Cochlearia officinalis* L. (Koch, Hurka, & Mummenhoff, 1996) and is a habitat specialist of calcareous springs with continuous water supply, small rivers, or drainage ditches and occurs in open calcareous fens, woodland clearings, and shaded woodland springs (Abs, 1999). The species is considered as highly endangered and is legally protected by law (Fischer et al., 2003).

*Cochlearia bavarica* is a perennial, monocarpic herbaceous plant species (Paschke, Bernasconi, & Schmid, 2003) with a sporophytic self‐incompatibility system (Fischer et al., 2003). Plants flower from May to June, and the ellipsoid fruits are 5–8 mm long and contain two to six brown or reddish‐brown seeds (Vogt, 1985). The species is pollinated by flies, bumblebees, other bees, or small moths (Paschke, Abs, & Schmid, 2002b; Paschke et al., 2003). Vegetative reproduction plays no major role because daughter rosettes are only found in the immediate vicinity of parent plants (Paschke et al., 2002b).

### 2.2 Study design and sampled populations

**Figure 1:**
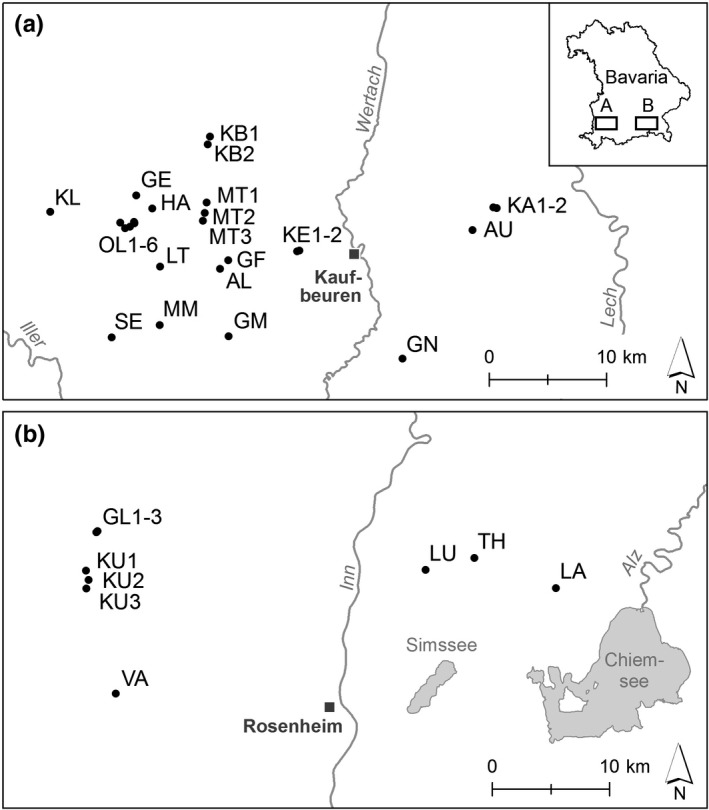
Geographic position of the analyzed populations of *Cochlearia bavarica* in Swabia (a) and Upper Bavaria (b)

In this study, we analyzed all 32 actually existing populations of *C. bavarica* (Table 1). Twenty‐four populations are located in Swabia, and eight populations in Upper Bavaria (Figure 1). Within the two regions, populations of *C. bavarica* are strongly isolated. However, single populations often consist of several subpopulations with a distance of less than 200 m in between. Genetic variation was therefore exemplarily analyzed within and among each three subpopulations in one population from Swabia and one population from Upper Bavaria. For molecular analysis, rosette leaves were collected in situ from fifteen individuals per population or subpopulation. In total, fresh leaf material of 517 individuals was sampled and dried in teabags over silica gel. Population size was obtained from the monitoring regularly conducted in the conservation projects and ranged from 6 up to 102,500 individuals (Table 1).

**Table 1:** Number, geographic location, and names of the analyzed populations in Swabia and Upper Bavaria. Subpopulations are displayed indented. Also specified are population label, number of analyzed individuals (*n*), and the population size (PS). Furthermore, genetic variation measures as Nei's gene diversity (GD), Shannon's information index (SI), and percentage of polymorphic bands (PB). Standard errors are given for mean values

No.	Region	Population (subpopulation)	Label	*n*	PS	GD	SI	PB
1	Swabia	Klessen	KL	15	600	0.1198	0.1769	32.83
2	Swabia	Ollarzried‐Daßberg	OL1	15	300	0.1255	0.1842	32.32
3	Swabia	Ollarzried‐Boschach	OL2	15	1,500	0.1280	0.1879	33.84
4	Swabia	Ollarzried‐Mitte	OL3	15	50	0.1239	0.1824	32.32
	Swabia	Ollarzried‐Höhe	OL	15	4,300	0.1275	0.1883	33.84
5	Swabia	‐Höhe 1	OL4	15	1,800	0.0968	0.1423	25.25
6	Swabia	‐Höhe 2	OL5	15	2,000	0.1219	0.1795	31.82
7	Swabia	‐Höhe 3	OL6	15	500	0.1213	0.1774	31.31
8	Swabia	Seebach	SE	6	6	0.0996	0.1421	22.73
9	Swabia	Grub‐Eheim	GE	15	7,500	0.0971	0.1416	24.75
10	Swabia	Hatzleberg	HA	15	200	0.1139	0.1676	30.81
11	Swabia	Liebenthann	LT	15	500	0.1022	0.1521	28.28
12	Swabia	Immenthal	MM	9	15	0.1015	0.1490	26.77
13	Swabia	Katzbrui‐Mariengrotte	KB1	15	95,000	0.1199	0.1759	31.31
14	Swabia	Katzbrui‐Mühle	KB2	15	7,500	0.0704	0.1053	20.71
15	Swabia	Mindeltal‐Schönlings	MT1	15	6,000	0.1141	0.1660	29.29
16	Swabia	Mindeltal‐Reichartsried	MT2	15	1,500	0.1087	0.1589	27.78
17	Swabia	Mindeltal‐Mayers	MT3	15	5,500	0.1115	0.1656	30.30
18	Swabia	Algers	AL	15	7,000	0.0950	0.1410	26.77
19	Swabia	Gfäll	GF	15	100	0.1108	0.1616	28.28
20	Swabia	Gillenmoos	GM	14	3,000	0.1140	0.1655	28.79
21	Swabia	Kemnath 1	KE1	15	8,500	0.1004	0.1465	25.76
22	Swabia	Kemnath 2	KE2	15	—	0.0931	0.1375	25.25
23	Swabia	Gennachquelle	GN	14	15	0.1261	0.1856	32.83
24	Swabia	Aufkirch	AU	6	7	0.0860	0.1243	20.71
25	Swabia	Kaltental 1	KA1	15	—	0.1074	0.1567	27.27
26	Swabia	Kaltental 2	KA2	15	15,000	0.0822	0.1212	22.73
	Mean all populations of Swabia					0.1074	0.1577	28.18
	Standard error					±0.0031	±0.0046	±0.81
	Upper Bavaria	Glonnquellen	GL	15	2,100	0.1057	0.1572	29.8
27	Upper Bavaria	‐Glonn 1	GL1	15	500	0.1101	0.1625	29.29
28	Upper Bavaria	‐Glonn 2	GL2	15	1,400	0.0934	0.139	26.26
29	Upper Bavaria	‐Glonn 3	GL3	15	200	0.0942	0.1397	26.26
30	Upper Bavaria	Kupferbachtal 1	KU1	15	500	0.0992	0.1456	26.26
31	Upper Bavaria	Kupferbachtal 2	KU2	15	1,000	0.0969	0.1423	26.77
32	Upper Bavaria	Kupferbachtal 3	KU3	15	8,000	0.1149	0.1691	30.81
33	Upper Bavaria	Vagen	VA	15	600	0.1038	0.1541	28.79
34	Upper Bavaria	Lungham	LU	15	2,000	0.1182	0.174	31.31
35	Upper Bavaria	Thalham	TH	15	1,000	0.1093	0.161	30.81
36	Upper Bavaria	Laubensee	LA	15	50	0.0746	0.1133	23.23
	Mean all populations of Upper Bavaria					0.1028	0.1521	28.47
	Standard error					±0.0048	±0.0067	±1.0011

### 2.3 Molecular analyses

Genetic variation was assessed using genome‐wide genotyping with AFLPs, amplified fragment length polymorphisms (Vos et al., 1995). DNA was isolated from silica gel dried plant material applying the cetyltrimethylammonium bromide method by Rogers and Bendich (1994) in an adaption by Reisch (2007). Concentration of genomic DNA was measured with a spectrophotometer, and every sample was diluted with water to a concentration of 7.8 ng/μl. The AFLP procedure was conducted in accordance with the protocol from Beckman Coulter as described before (Bylebyl, Poschlod, & Reisch, 2008; Reisch, 2008).

Double‐strand DNA adapters were produced by adding equal volumes of both single strands of *Eco*RI and *Mse*I adaptors (Biomers) in a 0.2‐ml reaction vessel, heating for 5 min at 95°C with a final 10‐min step at 25°C.

Digestion of 6.4 μl of genomic DNA (7.8 ng/μl) and ligation of DNA adaptors were performed by adding 3.6 μl of a core mix consisting of 2.5 U *Eco*RI (Thermo Scientific), 2.5 U *Mse*I (Thermo Scientific), 0.1 μmol/L *Eco*RI and 1 μmol/L *Mse*I adapter pair, 0.5 U T4 DNA ligase with its corresponding buffer (Thermo Scientific), 0.05 mol/L NaCl and 0.5 μg BSA (BioLabs/NBA), and a following incubation for 2 hr at 37°C and a subsequent enzyme denaturation step at 70°C for 15 min. The products were diluted 10‐fold with 1:10 TE buffer (20 mmol/L Tris‐HCl, pH 8.0; 0.1 mmol/L EDTA, pH 8.0).

In the preselective amplification, a reaction volume of 5 μl containing the diluted DNA restriction–ligation product, preselective *Eco*RI and *Mse*I primers (Biomers) with a single selective nucleotide (*Mse*I‐C and *Eco*RI‐A) and an AFLP core mix consisting of 1× Buffer S, 0.2 mmol/L dNTPs, and 1.25 U Taq‐Polymerase (PeqLab) were amplified under the chosen parameters: 2 min at 94°C; 30 cycles of 20 s denaturation at 94°C followed by 30 s annealing at 56°C and 2 min elongation at 72°C; finally 2 min at 72°C ended the elongation period; 30 min at 60°C and a cool down to 4°C completed the PCR run. After this, the products were diluted 20‐fold with 1:10 TE buffer for DNA.

For selective amplification, primers with three selective nucleotides were used. *Eco*RI primers were labeled with three different fluorescent dyes for fragment detection (Beckman dye D2, D3, and D4). After an extensive primer screening with eight randomly selected individuals, six primer combinations were chosen for further analysis: *Mse*I‐CTC/*Eco*RI‐AGC and *Mse*I‐CAC/*Eco*RI‐AAC (D2), *Mse*I‐CAA/*Eco*RI‐AAG and *Mse*I‐CAG/*Eco*RI‐AAG (D3), *Mse*I‐CTG/*Eco*RI‐ACT and *Mse*I‐CTA/*Eco*RI‐ACA (D4).

Selective amplifications were performed in a reaction volume of 5 μl containing an AFLP core mix (1× Buffer S, 0.2 mmol/L dNTPs), 1.25 U Taq‐Polymerase (PeqLab), 0.05 μmol/L selective *Eco*RI (Biomers), 0.25 μmol/L *Mse*I (Biomers) primers, and 0.75 μl diluted preselective amplification product. The PCR run started with 2 min at 94°C; then 10 cycles of 20 s denaturation at 94°C, 30 s annealing at 66°C (temperature was reduced every subsequent step by 1°C), and 2 min elongation at 72°C; then additional 25 cycles of 20 s denaturation at 94°C, 30 s annealing at 56°C, and 2 min elongation at 72°C, completed by a following 30 min step at 60°C and a cool down to 4°C.

Selective PCR products were diluted with 5 μl (D2) and with 20 μl (D4) 1xTE buffer for DNA.

Then, 5 μl amplified selective PCR product (of each D2, D3, and D4) was added to a stop solution, consisting of 2 μl sodium acetate (3 mol/L, pH 5.2), 2 μl Na_2_EDTA (100 mmol/L, pH 8), and 1 μl glycogen (Roche). Participation of DNA took place by adding 60 μl of ice‐cold ethanol (96%; −20°C), an immediate shaking and subsequent centrifugation for 20 min at 14,000 g at 4°C. The pelleted DNA was washed once by adding 200 μl of ice‐cold ethanol (70%; −20°C) and again centrifugation for 20 min at 14,000 g at 4°C. Afterward, the pelleted DNA was vacuum dried in a vacuum concentrator (Eppendorf) and dissolved in a mixture of 24.8 μl sample loading solution (Beckman Coulter) and 0.2 μl DNA Size Standard 400 (Beckman Coulter).

According to fragment size, the fluorescence‐labeled selective PCR products were separated by capillary gel electrophoresis on an automated sequencer (GenomeLab GeXP, Beckmann Coulter), and results were examined with DNA Size Standard 400 using the GeXP software (Beckman Coulter). For further investigations, results were exported as synthetic gel files (.crv), and the fragment pattern of every single individual was analyzed using the software Bionumerics 4.6 (Applied Maths, Kortrijk, Belgium): Each strong and clearly defined fragment was taken into account as either present or absent.

Samples with no clear banding pattern were repeated. Only three samples of *C. bavarica* had to be excluded from the analyses, due to amplification problems.

For quality control of the AFLP procedure, 10% of all analyzed samples were replicated twice and a genotyping error rate was calculated, according to Bonin et al. (2004), which was 3.2%.

### 2.4 Statistical analysis

Employing the software Bionumerics 4.6, a binary (0/1) matrix was created for statistical analyses. If present, fragments of a given length were detected as 1 and in the case of absence as 0. Using the matrix, genetic diversity within each population and subpopulation was calculated as the percentage of polymorphic bands (PB), as Shannon's information index SI = Σ(p_*i*_)ln(p_*i*_) and Nei's gene diversity (GD) *H* = 1 − Σ(p_*i*_)^2^, where p_*i*_ represents the allele frequency, by using the software PopGene 32 (Yeh, Yang, Boyle, Ye, & Mao, 1997). A Mann–Whitney *U* test was used to test for significant differences in genetic diversity between regions applying the software IBM Statistics 22 for Windows (IBM Corp). Spearman's rank correlation coefficient was calculated to test the impact of population size on genetic diversity.

Hierarchical analyses of molecular variance, AMOVA (Excoffier, Smouse, & Quattro, 1992), were conducted with the software GenAlEx 6.41 (Peakall & Smouse, 2006). Thus, genetic differentiation within and among subpopulations, populations, and between regions was investigated in two‐ and three‐level AMOVAs.

Correlation between genetic distances (Φ_PT_ values calculated in the AMOVA) and geographic distances among populations was tested in a Mantel test with 999 permutations (Mantel, 1967).

Genetic distances among populations were calculated as Nei's distance (Ds) following Lynch and Milligan (1994) with nonuniform prior distribution of allele frequencies in the program AFLPsurv (Vekemans, 2002). Based on these Ds distances, a consensus Neighbor‐Net graph was calculated applying the software SplitsTree 4.14.4 (Huson & Bryant, 2006). Additionally, distance matrices generated by bootstrapping (1,000 bootstrap replicates were performed) were written in AFLPsurv, too. The files were used as input for the NEIGHBOR and CONSENSE procedures from the PHYLIP software package version 3.695 (Felsenstein, 1993) to obtain bootstrap support values. Bootstrap values higher than 70% were plotted in the Neighbor‐Net graph.

Genetic relatedness of individuals was analyzed in the software MVSP version 3.12f (Kovach, 2007) using principal coordinate analyses (PCoA) based on interindividual Bray–Curtis similarities.

Moreover, a Bayesian cluster analysis was calculated with the program Structure version 2.3.4 (Pritchard, Stephens, & Donnelly, 2000; Pritchard, Wen, & Falush, 2007) to infer population structure in the data set and assign individuals into groups. It is assumed that the data set consists of an unknown number of *K* groups. Every single group is characterized by a set of allele frequencies at each locus, and samples from the data set are assigned randomly to groups. The number of groups was calculated using 10,000 Markov Chain Monte Carlo simulations with a burn‐in period of 100,000 iterations. Analyses for the predefined value of *K* were run 20 times per *K* = 1–40 (Falush, Stephens, & Pritchard, 2003, 2007). The program Structure Harvester (Earl & Vonholdt, 2012) was used to summarize results. Group assignment was an ad hoc quantity procedure calculating Δ*K* (Evanno, Regnaut, & Goudet, 2005). The best estimate of *K* for the data set was defined according to the model which gave the consistent results for multiple runs and the highest probability of the data.

## 3 RESULTS

### 3.1 AFLP banding and genetic diversity

Amplified fragment length polymorphisms analysis resulted in 198 fragments. No identical genotypes were detected. Furthermore, there were four bands private to the populations from Swabia and eleven bands were found only in populations from Upper Bavaria; 75.76% of the fragments were polymorphic.

In populations from Swabia, Nei's GD ranged from 0.07 to 0.13 (mean 0.11), Shannon's information index (SI) from 0.11 to 0.19 (mean 0.16), and the percentage of PB from 20.71 to 33.34 (mean 28.18). The highest level of diversity was found in population Ollarzried‐Boschach and the lowest in population Katzbrui‐Mühle (Table 1).

Similar results were found in populations from Upper Bavaria (Table 1). GD ranged from 0.08 to 0.12 (mean 0.10) and SI from 0.11 to 0.17 (mean 0.15). The percentage of polymorphic bands varied between 23.23 and 31.31 (mean 28.47). The highest level of diversity was found in population Lungham and the lowest in population Laubensee.

Populations from Swabia and Upper Bavaria did not differ significantly in genetic diversity, and the estimated population size was not correlated with genetic diversity (Spearman's rank correlation coefficient: *r*_GD_ = −.22, *p*_GD_ = .91).

### 3.2 Genetic differentiation

**Figure 2:**
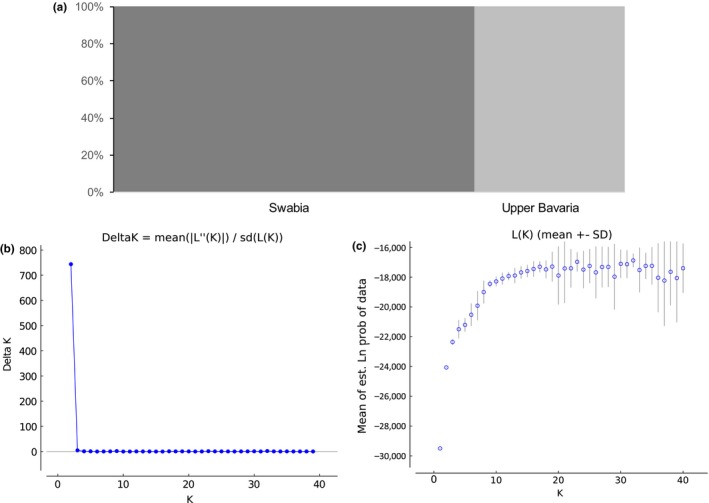
Results of the Bayesian cluster analysis. Populations were assigned to two groups according to the geographic regions Swabia and Upper Bavaria (a). Results of 20 runs for 1–40 possible groups to infer population structure with Bayesian clustering in STRUCTURE are shown in graph (b). Delta *K* is shown for each of the tested groups *K* = 1–40. Graph (c) shows LnP(*D*) variance for each of the tested groups

In the Bayesian cluster analysis, individuals were assigned to two groups (Δ*K* = 743.8) reflecting the regions Swabia and Upper Bavaria. For *K* = 2, outputs of all 20 iterations were identical (Figure 2a–c).

**Figure 3:**
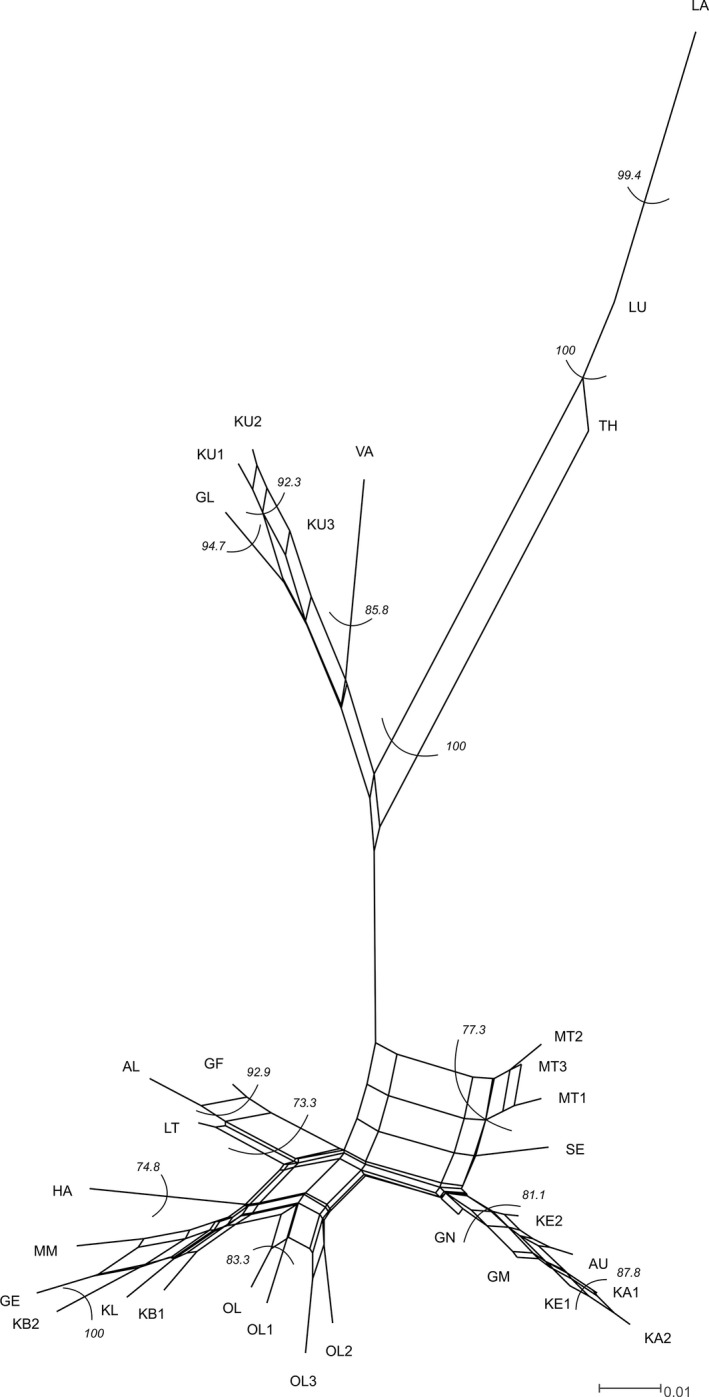
Consensus Neighbor‐Net of all *Cochlearia bavarica* populations based on the **amplified fragment length polymorphisms** data. Populations from Swabia and Upper Bavaria were clearly separated. Bootstrap values >70% are given in italics. The fit value is 93.47

In the Neighbor‐Net analysis, the studied populations were also assigned to these regions (Figure 3). Within Swabia, populations formed three groups: one comprised populations from the locations Hatzleberg (HA), Immenthal (MM), Grub‐Eheim (GE), Katzbrui (KB), Klessen (KL), and Ollarzried (OL); the second consisted of populations from Liebenthann (LT), Algers (AL), and Gfäll (GF). Populations from the locations Gennachquelle (GN), Gillenmoos (GM), Kemnath (KE), Kaltenthal (KA), Aufkirch (AU), Seebach (SE), and Mindeltal (MT) formed the third group. In Upper Bavaria, the populations Lungham (LU), Thalham (TH), and Laubensee (LA) were clearly separated from a second group, which comprised the populations from Glonnquellen (GL), Kupferbachtal (KU), and Vagen (VA).

**Figure 4:**
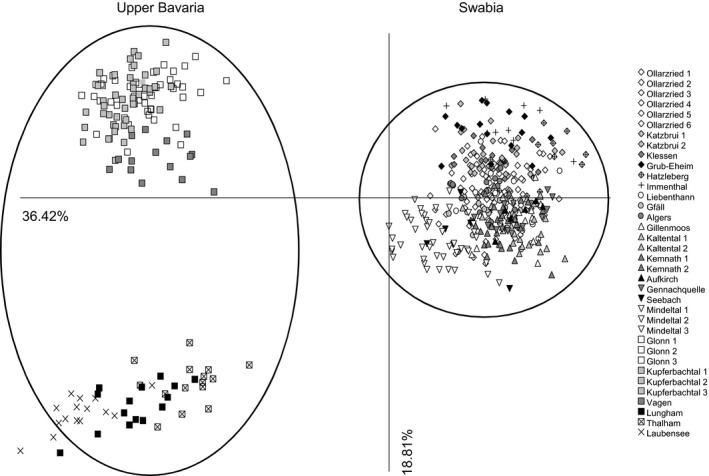
Principal coordinates analysis of all sampled individuals of *Cochlearia bavarica* from Swabia and Upper Bavaria based on amplified fragment length polymorphisms data. Axis 1 explains 36.34% of variance; axis 2 explains 18.81% of variance. Populations from Swabia and Upper Bavaria were clearly separated and formed two groups

**Figure 5:**
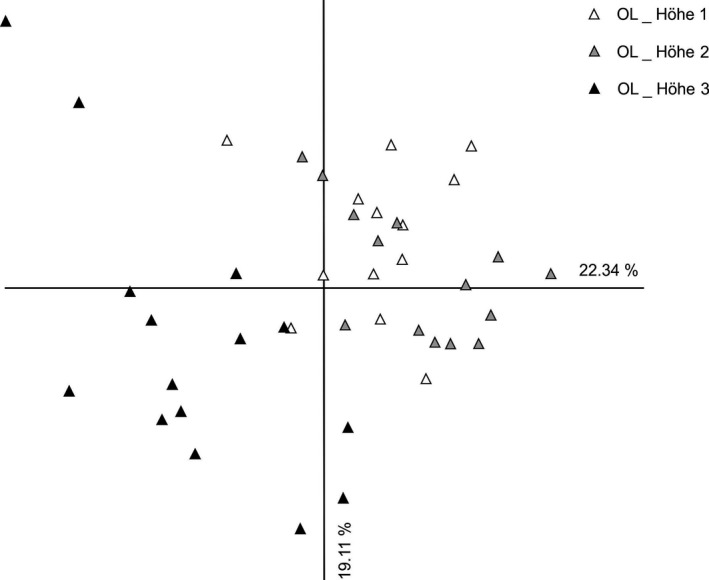
Principal coordinates analysis of sampled individuals of *Cochlearia bavarica* from Swabia based on amplified fragment length polymorphisms data. Axis 1 explains 22.34% of variance; axis 2 explains 19.11% of variance. No population grouping could be observed

**Figure 6:**
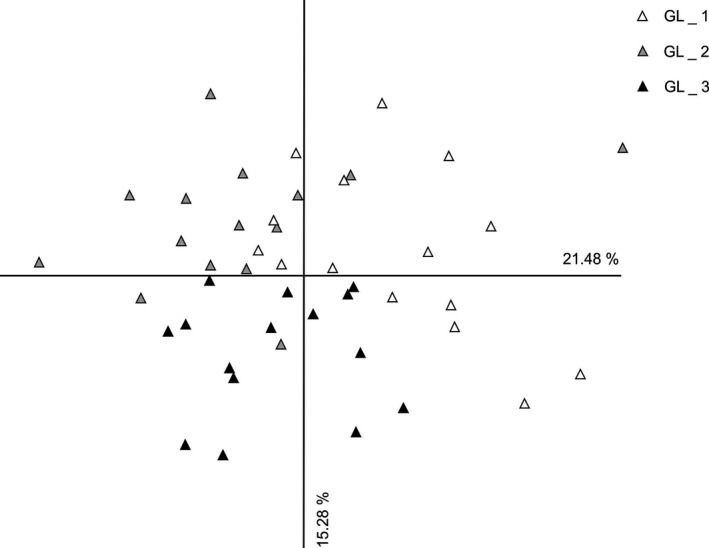
Principal coordinates analysis of sampled individuals of *Cochlearia bavarica* from Upper Bavaria based on **amplified fragment length polymorphisms** data. Axis 1 explains 21.48% of variance; axis 2 explains 15.28% of variance. No population grouping could be detected

The PCoA results were similar to the results from the Bayesian cluster analysis and the Neighbor‐Net analysis and also revealed a strong separation of individuals from Swabia and Upper Bavaria (Figure 4). At the subpopulation level, individuals from different subpopulations were mostly admixed in the two studied populations from Swabia (Figure 5) and Upper Bavaria (Figure 6). Only subpopulation Ollarzried Höhe 3 exhibited a slightly stronger level of differentiation.

In the three‐level AMOVA, we also observed a very strong genetic differentiation between the two study regions Swabia and Upper Bavaria with a Φ_PT_ value of 0.62 (Table 2). Within these regions, genetic differentiation among populations was also strong but weaker among populations from Swabia (Φ_PT_ = 0.38) than among populations from Upper Bavaria (Φ_PT_ = 0.51). Further analyses revealed only a low level of genetic differentiation among subpopulations in Swabia (Φ_PT_ = 0.13) and Upper Bavaria (Φ_PT_ = 0.12).

**Table 2:** Molecular variance within and among populations of *Cochlearia bavarica* calculated in different analyses of molecular variance based on 198 amplified fragment length polymorphisms fragments. Levels of significance are based on 999 iteration steps and are indicated by three asterisks (*p* < .001)

	*df*	SS	MS	%	ΦPT
Molecular variation between regions					
Among regions	1	1,515.19	1,515.19	34.02	0.62***
Among populations	30	3,011.66	100.39	27.49	
Within populations	422	3,813.43	9.04	38.49	
Molecular variation among populations within regions					
Swabia					
Among populations	23	1,949.35	84.75	37.57	0.38***
Within populations	310	2,807.03	9.055	62.43	
Upper Bavaria					
Among populations	7	1,062.31	151.76	51.44	0.51***
Within populations	112	1,006.4	8.99	48.56	
Molecular variation among subpopulations within populations					
Swabia—Ollarzried‐Höhe					
Among subpopulations	2	60.71	30.36	12.56	0.13***
Within subpopulations	42	404.13	9.62	87.44	
Upper Bavaria—Gollquellen					
Among subpopulations	2	52.89	26.44	12.1	0.12***
Within subpopulations	42	362.4	8.63	87.9	

*df*, degree of freedom; SS, sum of squares; MS, mean squares; %, proportion of genetic variability.

**Figure 7:**
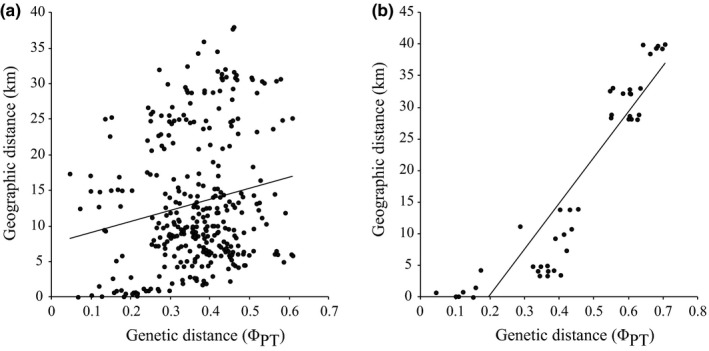
Correlation of genetic distance (Φ_PT_) and geographic distance (km) between populations and subpopulations (Mantel test) for the populations in Swabia (a, *r* = .18, *p* = .02) and the populations in Upper Bavaria (b, *r* = .92, *p* = .001) of *Cochlearia bavarica*

A Mantel test including all populations revealed significant correlation between pairwise genetic distances and geographic distances (*r* = .80, *p* = .001). Additional Mantel tests have been implemented for each distribution area (Figure 7). In Swabia, we found only a weak but significant correlation of genetic distance with spatial distance (*r* = .18, *p* = .02). In contrast, this correlation was very strong for populations in Upper Bavaria (*r* = .92, *p* = .001).

## 4 DISCUSSION

### 4.1 Genetic diversity

In our study, genetic diversity within the analyzed populations of *C. bavarica* was low but within the range observed for species with similar traits (Hamrick & Godt, 1996; Nybom, 2004; Nybom & Bartish, 2000). Nei's GD of *C. bavarica* was on average 0.10 and, therefore, even slightly lower than previously reported for other rare species (0.12) in a literature survey based on more than 150 plant species (Reisch & Bernhardt‐Römermann, 2014).

Isolation of predominantly small populations is the most important reason for reduced levels of genetic diversity in populations of rare plant species. Indeed, populations of *C. bavarica* are strongly isolated (Fischer et al., 2003), with large geographic distances in between. Moreover, many populations are surrounded by dense forests, which enhances isolation because the forests represent effective barriers for pollinators (Paschke et al., 2002b). Although the species is considered as self‐incompatible, Fischer et al. (2003) found a certain degree of self‐compatibility. With increasing distance between populations gene flow decreases, which means that self‐pollination and mating events between related individuals may become more frequent and decrease the level of genetic diversity.

In the last three decades, many populations of *C. bavarica* disappeared due to habitat degradation and nutrient enrichment, which resulted in a proceeding fragmentation. This process of habitat fragmentation is a general threat to biodiversity, reducing species richness within small and isolated habitat patches (Fahrig, 2003). However, fragmentation also affects genetic diversity because population size decreases, and gene flow among small and isolated remnant populations is strongly reduced (Vitousek, 1994). The exchange of pollen and seeds between populations is restricted (Honnay et al., 2006), and consequently, genetic diversity within populations is declining. This process of genetic erosion (Luijten et al., 2000; Oostermeijer, 1996; Young et al., 1996) reduces in the long term the adaptability to changing environmental conditions (Booy et al., 2000; Heywood, 1991) and may even cause extinction (Frankham, 2005).

Even though the level of fragmentation and isolation is stronger in Upper Bavaria than in Swabia, we observed in our study no significant differences in genetic diversity between populations from the two study regions. This is most likely due to the fact that although populations are more frequent in Swabia than in Upper Bavaria, the populations are nevertheless strongly isolated. Differences in frequency seem to be too small to result in different levels of genetic diversity.

The positive relationship between population size and genetic diversity has been reported in numerous studies (Fischer & Matthies, 1998; Frankham, 1996; Godt, Johnson, & Hamrick, 1996; Hamrick & Godt, 1990; Leimu, Mutikainen, Koricheva, & Fischer, 2006). However, we observed no significant positive correlation between these two parameters. Previous investigations revealed higher levels of allozyme variation (Paschke et al., 2002b) in larger than in smaller populations of *C. bavarica*. However, this study was based on data collected 15 years ago, and the populations of *C. bavarica* further declined since then. This may be the reason why our results differ from the previous study on allozyme variation. Indeed, many investigations revealed no correlation between population size and genetic variation mainly due to lag effects or long‐term survival under highly fragmented conditions (Honnay & Jacquemyn, 2007; Kuss, Pluess, Aegisdottir, & Stocklin, 2008).

### 4.2 Genetic differentiation

With a Φ_PT_ of 0.62, our study revealed a high level of genetic differentiation between populations of *C. bavarica*. The level of differentiation is much higher than previously reported (Reisch & Bernhardt‐Römermann, 2014) for other rare species (Φ_PT_ of 0.34) and reflects the strong fragmentation and isolation of *C. bavarica*. Generally, genetic differentiation between populations depends on the interplay of gene flow and drift (Slatkin, 1987). Under highly fragmented and isolated conditions, gene flow decreases, while genetic differentiation due to drift increases (Vitousek, 1994). In the case of *C. bavarica*, this process may be enhanced by potential self‐pollination further increasing genetic differentiation (Reisch & Bernhardt‐Römermann, 2014).

However, the level of genetic differentiation varied in our study strongly between different spatial scales. Considering the whole distribution range, we found a strong differentiation between the two regions Swabia and Upper Bavaria. This observation is supported by previous studies revealing a number of alleles being characteristic for either Swabian or Upper Bavarian populations (Koch, Huthmann, & Hurka, 1998; Paschke et al., 2002b). Within both regions, we observed a significant correlation of genetic and geographic distances between populations in the Mantel test. However, the correlation was weaker in Swabia than in Upper Bavaria. In Swabia, geographically adjacent populations were not necessarily genetically more similar to each other than geographically more distant populations as shown for the population Seebach and the populations from Mindeltal or the populations Immenthal, Katzbrui, Klessen, and Grub‐Eheim. In contrast, we observed a clear pattern of isolation by distance in Upper Bavaria. Furthermore, genetic differentiation between populations was lower in Swabia than in Upper Bavaria. This corresponds to the results of the Mantel test and can be ascribed to the fact that populations are and may also have previously been more frequent in Swabia than in Upper Bavaria. Historical gene flow may, therefore, have been stronger among the more frequent populations in Swabia and more limited among the populations from Upper Bavaria. Referring to the genetic structure within populations, we observed only limited differentiation between subpopulations, which were less than 200 m distant to each other. The analysis of molecular variance revealed only low levels of genetic differentiation, and the cluster analyses indicated the admixture of individuals. Obviously, pollination seems to be hardly limited at this distance, which is supported by previous studies providing evidence that gene flow by pollen is normally restricted to the nearest vicinity of plant populations to distances of less than one kilometer (Aavik, Holderegger, & Bolliger, 2014; Kwak, Velterop, & van Andel, 1998).

## 5 CONCLUSIONS WITH RESPECT TO CONSERVATION

The aim of this study was to increase the success of future population reintroduction and reinforcement, by providing data to avoid negative effects of inbreeding and outbreeding and to preserve the natural genetic pattern of the species. However, it should be kept in mind that every reintroduction project with its species is unique (Guerrant & Kaye, 2007) and that generalizations are, therefore, limited. Nevertheless, it is possible to draw conclusions for a successful reintroduction of *C. bavarica* from our study.

It has been demonstrated that reintroduction success can be enhanced by using plant material from large and stable source populations (Godefroid et al., 2011). In the case of *C. bavarica*, large populations were not necessarily genetically most variable. Since bottlenecks, inbreeding and drift can be avoided best by taking plant material from populations with a high level of genetic diversity we suggest, therefore, to use rather highly variable than large source populations for the reintroduction or the reinforcement of *C. bavarica* such as the population Ollarzried‐Boschach in Swabia or the population Lungham in Upper Bavaria. Within these populations, plant material should be collected where possible from 50 up to 200 individuals of different age and size classes (Lauterbach, 2013) all over the population to sample genetic diversity representatively (Brown & Briggs, 1991). Moreover, reintroduction success can be improved by acting at a large scale (Frankham et al., 2002). In previous studies, 500 up to 5,000 individuals have proven as a suitable number of individuals for successful reintroduction (Given, 1994; Pavlik, 1996; Reed, 2005). We strongly recommend, therefore, using a large number of individuals for the planned reintroduction of the species.

Although mixing plant material from multiple source populations has been successfully used for reintroduction (Godefroid et al., 2011; Guerrant & Kaye, 2007; Maschinski, Wright, Koptur, & Pinto‐Torres, 2013), because using large numbers of unrelated individuals contributes to a large and diverse gene pool (Vergeer et al., 2005), this approach should be handled with care due the risk of outbreeding depression, which reduces fitness and performance (Bischoff et al., 2006; Fischer & Matthies, 1998; Keller et al., 2000; Krauss et al., 2005; Mijnsbrugge et al., 2010; Montalvo & Ellstrand, 2000, 2001). Furthermore, mixing material from different source populations should be avoided if the spatial genetic pattern of a species should be preserved (Gordon, 1994). *Cochlearia bavarica* exhibited a very distinct geographic pattern of genetic variation, and we would, therefore, strongly advise against using multiple source populations for reintroductions and population reinforcement. Instead, we suggest a graduated procedure for the reintroduction of the species, considering the observed pattern of genetic variation. Because our study revealed a very strong level of genetic differentiation between Swabia and Upper Bavaria, the exchange of plant material between these two study regions should be completely avoided. Within these regions, we detected different patterns of genetic variation. Although we found a clear pattern of isolation by distance in Upper Bavaria, the situation was more idiosyncratic in Swabia. Most likely due to historic gene flow, geographically adjacent populations were not necessarily genetically similar to each other. We suggest, therefore, different approaches for the two regions. In Upper Bavaria for reintroduction, plant material should preferably be used from closely located and, therefore, genetically most similar populations to avoid outbreeding. In Swabia, two different approaches are conceivable: if conservationists decide to preserve the current pattern of genetic variation, plant material for reintroduction should be taken from the genetically most similar population, and if they decide that the present pattern should not be kept since it resulted from former gene flow anyway, plant material should be used from the most variable source population. At the subpopulation level, we detected only a low level of differentiation with a high degree of admixture between subpopulations due to gene flow. The transfer of plant material between subpopulations should, therefore, be possible without changing the natural genetic pattern of the species and without the risk of outbreeding.

It has already been demonstrated that a specific management of the reintroduction sites increases the reintroduction success. Moreover, a reliable and continuous monitoring allows the evaluation of population reintroduction success (Godefroid et al., 2011). Therefore, we strongly recommend a continuous long‐term monitoring of the reintroduced *C. bavarica* individuals and a thorough management of the reintroduction sites.

## CONFLICT OF INTEREST

The authors declare that they have no conflict of interest.

## AUTHOR CONTRIBUTIONS

C.R. conceived and designed the study. F.K. collected the data and performed the analyses. Both authors contributed to manuscript writing.
